# Nonalcoholic Hepatic Steatosis Is a Strong Predictor of High-Risk Coronary-Artery Plaques as Determined by Multidetector CT

**DOI:** 10.1371/journal.pone.0131138

**Published:** 2015-06-30

**Authors:** Kazuhiro Osawa, Toru Miyoshi, Kentarou Yamauchi, Yasushi Koyama, Kazufumi Nakamura, Shuhei Sato, Susumu Kanazawa, Hiroshi Ito

**Affiliations:** 1 Department of Cardiovascular Medicine, Okayama University Graduate School of Medicine, Dentistry and Pharmaceutical Sciences, Okayama, Japan; 2 Department of Radiology, Okayama University Hospital, Okayama, Japan; 3 Cardiovascular Center, Sakurabashi Watanabe Hospital, Osaka, Japan; 4 Department of Radiology, Okayama University Graduate School of Medicine, Dentistry and Pharmaceutical Sciences, Okayama, Japan; University of Verona, Ospedale Civile Maggiore, ITALY

## Abstract

**Background:**

Nonalcoholic fatty liver disease is associated with a risk of coronary artery disease (e.g., diabetes mellitus, dyslipidemia, metabolic syndrome). We evaluated whether nonalcoholic hepatic steatosis is associated with high-risk plaques as assessed by multidetector computed tomography (CT).

**Methods:**

This retrospective study involved 414 participants suspected of having coronary artery disease. Nonalcoholic hepatic steatosis was defined as a liver-to-spleen fat ratio of <1.0 and the presence and appropriate characteristics of coronary-artery plaques as assessed by coronary CT angiography. High-risk plaques were identified, as were low-density plaques, positive remodeling, and spotty calcification.

**Results:**

Compared with patients who did not have nonalcoholic hepatic steatosis, patients with nonalcoholic hepatic steatosis had more low-density plaques (21% *vs*. 44%, p<0.01), positive remodeling (41% *vs*. 58%, p = 0.01), and spotty calcification (12% *vs*. 36%, p<0.01). The number of high-risk plaques in patients with nonalcoholic hepatic steatosis was greater than in those without nonalcoholic hepatic steatosis (p<0.01). Patients with nonalcoholic hepatic steatosis were more likely to have high-risk plaques than were those with only an elevated level of visceral adipose tissue (≥86 cm^2^; 35% *vs*. 16%, p<0.01). Multivariate analyses that included nonalcoholic hepatic steatosis, amount of visceral adipose tissue, and the presence/absence of traditional risk factors demonstrated that nonalcoholic hepatic steatosis was an independent predictor of high-risk plaques (odds ratio: 4.60; 95% confidence interval: 1.94–9.07, p<0.01).

**Conclusions:**

Diagnosis of nonalcoholic hepatic steatosis may be of value when assessing the risk of coronary artery disease.

## Introduction

Nonalcoholic fatty liver disease (NAFLD) is the most frequent cause of abnormal liver function, as documented in an urban population in the USA [1]. NAFLD is associated with several systemic diseases (e.g., visceral obesity, type-2 diabetes mellitus, dyslipidemia, hypertension), all of which are typical manifestations of the metabolic syndrome [2]. Association of NAFLD with such diseases has been attributed to cardiovascular disease [3–7]. Liver biopsies have been considered to be the “gold standard” for the diagnosis of NAFLD [8]. However, the imaging modality multi-detector computed tomography (MDCT) can provide a more accurate assessment of NAFLD than liver biopsies because it yields reproducible results, and NAFLD is closely correlated with fat accumulation in the liver [9,10].

Visceral adipose tissue (VAT) has also been shown to be independently associated with metabolic syndrome, diabetes mellitus, coronary artery disease (CAD), and CAD-related deaths [2]. The extent of VAT has been employed as a clinical measure of the risk of obesity. Recently, we reported the significant influence of larger VAT on the morphology of coronary-artery plaques, especially for high-risk plaques, which often contribute to acute coronary syndrome [11]. However, NAFLD has also been associated with coronary plaques [4] even in the arteries of patients who do not have metabolic syndrome [12]. NAFLD has also been associated with atherogenic dyslipidemia, hyperglycemia, and hypertension, regardless of the amount of VAT [13].

Before the present study, the impact of NAFLD on the presence and characteristics of coronary-artery plaques had not been investigated in detail. The purpose of this study was to determine whether NAFLD is associated with high-risk plaques as assessed by MDCT. However, the diagnosis of NAFLD by liver biopsy is difficult to apply for all patients undergoing MDCT. In the present study, nonalcoholic hepatic steatosis as defined by MDCT was evaluated for the association between NAFLD and high-risk coronary plaques.

## Methods

### Study population

**Fig 1:**
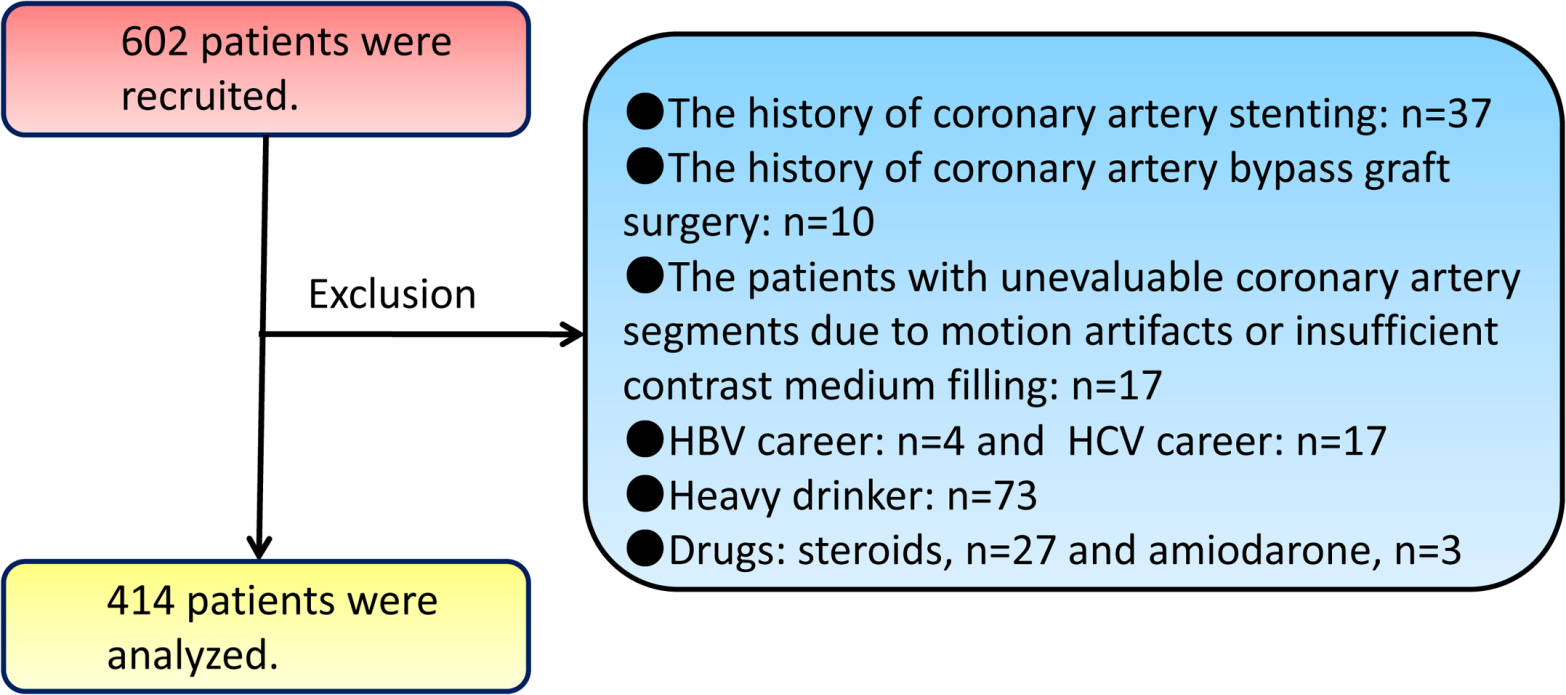
Patient acceptance into the study. HBV, hepatitis B virus; HCV, hepatitis C virus.

The initial population surveyed was 602 patients who had been evaluated consecutively at Okayama University Hospital (Okayama, Japan) between August 2011 and March 2013 using 64-slice MDCT because of suspected CAD. Patients with implanted coronary-artery stents (n = 37) or who had coronary artery bypass graft surgery (n = 10) were excluded. Patients who had unevaluated coronary-artery segments owing to motion artifacts or inadequate contrast-medium filling were also excluded (n = 17). In addition, individuals who consumed >20 g of alcohol per day (n = 73), those with known liver disease (carriers of the hepatitis B virus or hepatitis C virus, n = 21), or who were currently using oral corticosteroids (n = 27) and/or amiodarone (n = 3) were excluded. Such exclusion criteria left 414 subjects for assessment (Fig 1).

### Ethics statement

The present study was approved by the Ethics Committee of Okayama University Graduate School of Medicine, Dentistry, and Pharmaceutical Sciences (Okayama, Japan). This study was conducted according to the principles expressed in the Declaration of Helsinki. All patients provided written informed consent to be included in the study.

### MDCT

CT images were acquired using a SOMATOM Definition Flash instrument (Siemens Medical Solutions, Munich, Germany) as described previously [14]. A CT acquisition protocol using a test bolus was carried out at the level of the ascending aorta after administration of 10 ml of the contrast medium (Omnipaque 350; Daiichi Sankyo, Tokyo, Japan), then 15 ml of physiologic (0.9%) saline. A low-dose image was obtained every 1 s. The delay before formal imaging was calculated as the time to peak enhancement in the ascending aorta plus 3 s to ensure enhancement of the distal segments of the coronary arteries. For formal imaging, the initial injection time of the contrast agent was 12 s, and was followed by a second bolus of the contrast agent that had been diluted 1:1 with physiologic saline for an additional 8 s. Then, the contrast agent was “chased” with a bolus of physiologic saline (20 ml). The flow rate for all injections was equal to (body weight) × 0.07 ml/s.

### Coronary CT angiography analyses

**Fig 2:**
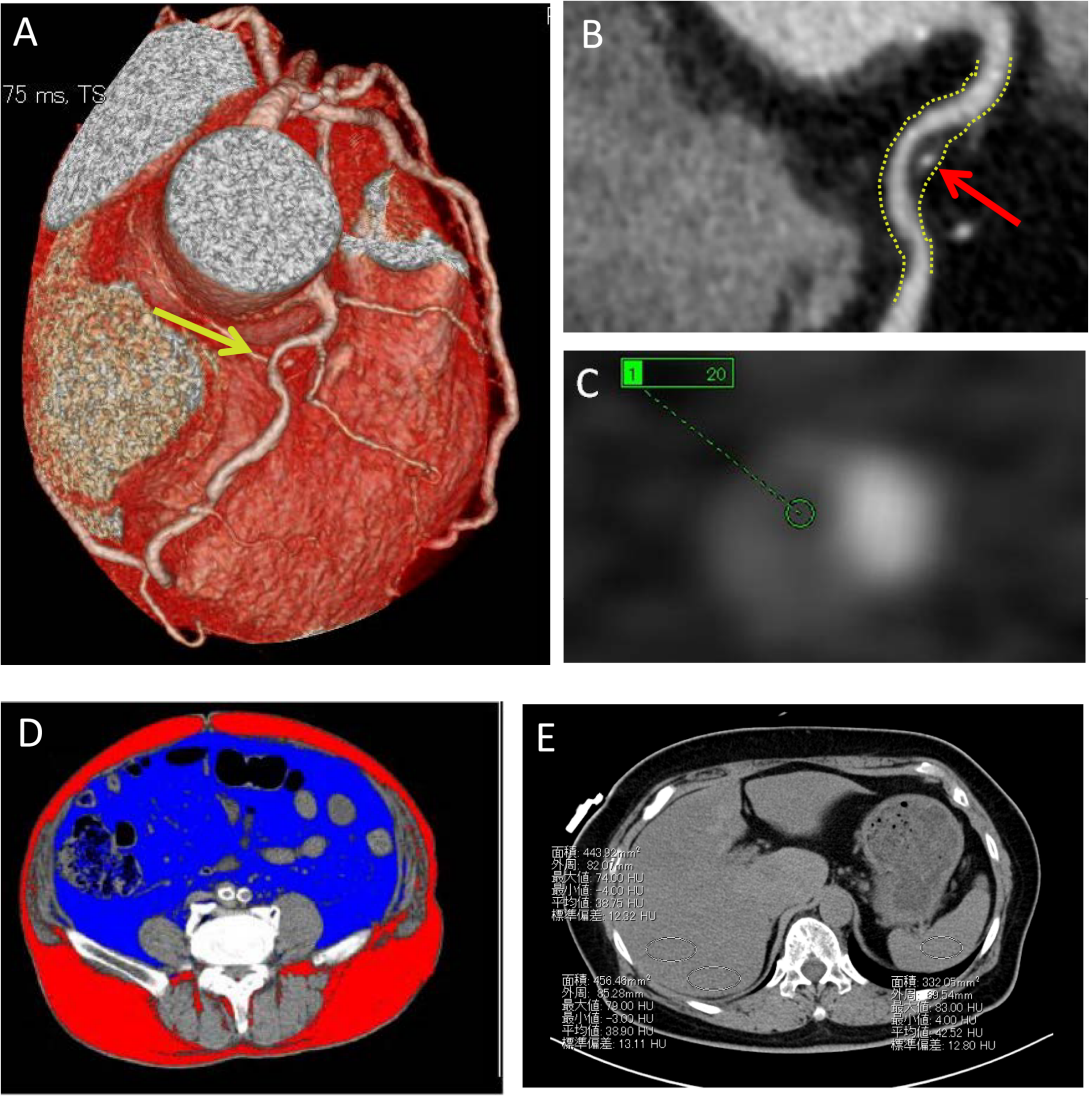
CT images of a coronary plaque, visceral adipose tissue, and liver. (A), Multidetector computed tomography imaging of a high-risk plaque with volume rendering; (B), curved multi-planar reconstruction image; and (C), an axial image. The yellow arrow in (A) denotes a high-risk plaque site. This lesion was positively remodeled as shown by comparison with a normal coronary segment proximal to the lesion (yellow dotted line in (B)). The red arrow in (B) denotes a region of spotty calcification, which is a low-density plaque of 20 HU. (D) CT of the liver of a patient with nonalcoholic hepatic steatosis. (E) CT image of a high area of visceral adipose tissue (220.4 cm^2^). Diffuse fat accumulation is observed in the liver in an unenhanced CT image with an L/S ratio of 0.74.

We used axial and curved multiplanar reformatted images to evaluate the morphology of coronary-artery plaques with commercially available cardiac reconstruction software (Virtual Place; Raijin, AZE Inc., Tokyo, Japan) (Fig 2A–2C) [15]. One experienced senior cardiologist and two senior CT technicians evaluated the images on a per-segment basis with 16 segments examined as described previously using the segment model developed by the American Heart Association [16]. Plaques were categorized as “calcified” (when the number of Hounsfield Units (HU) was >130), “non-calcified” (HU<130), or “low-density” (HU<50). We also assessed if coronary-artery remodeling had occurred using the ratio of the vessel diameter at the plaque site and a normal-appearing segment diameter proximal to the lesion (positive remodeling index >1.05). “Spotty” calcifications were defined as having a length (longitudinal direction of the vessel) and width (perpendicular to the longitudinal direction of the vessel) of calcification that were <3/2 and <2/3 of the vessel diameter, respectively. [11] We defined a plaque as “high-risk” when positive remodeling, low-density plaque, and spotty calcification had been completed.

### Measurements of abdominal tissue

Before cardiac imaging, an abdominal non-contrast CT scan was carried out at the umbilical level. Waist circumference was calculated automatically at this level (Fig 2D). VAT and subcutaneous adipose tissue were measured by a semi-automatic segmentation method. The attenuation limit for a fat-tissue deposit was defined as the interval within two standard deviations from the mean value for each individual. The muscular abdominal wall was traced manually to separate VAT from subcutaneous adipose tissue.

Hepatic and splenic Hounsfield attenuations were measured in the largest possible regions of interest (i.e., ≥1-cm^2^ areas that did not include large vessels or biliary structures) [9]. Regions of interest included two that were aligned with the anterior–posterior dimension of the right liver lobe and one aligned with the spleen. The ratio of hepatic-to-spleen attenuation (L/S ratio) was calculated by taking the mean HU measurement of the two right-liver-lobe regions of interest and dividing it by the spleen HU measurement (Fig 2E). We defined a L/S ratio <1.0 as the cutoff for a positive diagnosis of nonalcoholic hepatic steatosis [10].

### Statistical analysis

Categorical variables are the number of patients (percentage). Continuous variables are the mean ± the standard deviation (SD). Levels of triglyceride, high-sensitivity C-reactive protein (CRP), and Agatston scores are reported as medians (interquartile range). Differences between any two groups were evaluated using Pearson’s χ^2^ test for categorical variables and Student’s *t*-test for continuous variables. We defined “higher VAT” as >74 cm^2^. This cutoff value was determined according to analyses of receiver operator characteristic curves for discrimination of high-risk plaques. Multivariate logistic regression analyses were adjusted for age, sex, hypertension, dyslipidemia, diabetes mellitus, smoking, higher VAT, nonalcoholic hepatic steatosis, medication use. p<0.05 was considered significant. All statistical analyses were undertaken using SPSS v17.0 for Windows (IBM, Armonk, NY, USA).

## Results

All patients had suspected CAD and were diagnosed clinically before coronary CT angiography as follows: effort angina (n = 171), silent myocardial ischemia (n = 221), vasospastic angina (n = 15) and acute coronary syndrome (n = 7). Of the 414 participants (64 ± 15 years, 51% males), 64 (15%) had nonalcoholic hepatic steatosis. Compared with patients who did not have nonalcoholic hepatic steatosis, more patients with nonalcoholic hepatic steatosis were male, used dipeptidyl peptidase-4 inhibitors and had: a higher prevalence of hypertension and diabetes mellitus; higher levels of triglyceride and LDL-cholesterol; lower level of HDL-cholesterol; higher level of hemoglobin A1c; higher level of high-sensitivity CRP; (Table 1). With regard to adiposity, patients with nonalcoholic hepatic steatosis had a considerably greater body mass index, waist circumference, and areas of subcutaneous adipose tissue and VAT than those who did not have nonalcoholic hepatic steatosis.

**Table 1:** Characteristics of patients with and without nonalcoholic hepatic steatosis.

	Number of patients(n = 414)	Patients with nonalcoholic hepatic steatosis L/S < 1.0 (n = 64)	Patients without nonalcoholic hepatic steatosis L/S ≥ 1.0 (n = 350)	p
Age (years)	64 ± 15	62 ± 11	64 ± 15	0.43
Men, n (%)	210 (51)	42 (66)	168 (48)	<0.01
Body mass index (kg/m^2^)	23 ± 4	26 ± 4	23 ± 3	<0.01
Waist circumference (cm)	84 ± 11	92 ± 11	83 ± 10	<0.01
Hypertension, n (%)	242 (58)	53 (83)	189 (54)	<0.01
Dyslipidemia, n (%)	198 (48)	35 (55)	163 (47)	0.19
Diabetes mellitus, n (%)	118 (29)	31 (48)	87 (25)	<0.01
Currently smoking, n (%)	74 (18)	12 (19)	62 (18)	0.84
Metabolic syndrome, n (%)	83 (20)	31(48)	52 (15)	<0.01
Total cholesterol (mg/dl)	191 ± 34	200 ± 39	189 ± 36	0.03
Triglycerides (mg/dl)	111 (85)	158 (143)	104 (72)	<0.01
HDL-cholesterol (mg/dl)	57 ± 15	49 ± 12	59 ± 15	<0.01
LDL-cholesterol (mg/dl)	114 ± 32	122 ± 35	113 ± 31	0.04
Hemoglobin A1c (%)	6.2 ± 1.3	6.9 ± 1.7	6.1 ± 1.2	<0.01
Uric acid (mg/dl)	5.4 ± 1.5	6.1 ± 1.3	5.2 ± 1.5	<0.01
AST (IU/l)	25 ± 17	30 ± 16	24 ± 17	<0.01
ALT (IU/l)	25 ± 26	38 ± 27	22 ± 26	<0.01
γ-GTP (IU/l)	38 ± 50	51 ± 43	36 ± 51	0.04
Fasting plasma glucose (mg/dl)	102 ± 19	108 ± 19	100 ± 19	0.03
High-sensitivity CRP (mg/dl)	0.08 (0.13)	0.12 (0.14)	0.07 (0.13)	0.03
Subcutaneous adipose tissue (cm^2^)	146 ± 82	180 ± 80	139 ± 81	<0.01
Visceral adipose tissue (cm^2^)	92 ± 55	143 ± 62	83 ± 48	<0.01
Metabolic syndrome, n (%)	83 (20)	31 (48)	52 (15)	<0.01
Agatston score	10 (81)	25 (152)	9 (192)	0.64
Medications				
ACEI or ARB, n (%)	149 (36)	29 (45)	120 (34)	0.11
Calcium channel blocker, n (%)	121 (29)	21 (33)	100 (29)	0.54
Statin, n (%)	123 (30)	18 (28)	105 (30)	0.76
Sulfonylurea, n (%)	16 (4)	4 (6)	12 (3)	0.28
α-glucosidase inhibitor, n (%)	17 (4)	5 (8)	12 (3)	0.11
DPP-4 inhibitor, n (%)	27 (7)	12 (19)	15 (4)	<0.01
Insulin, n (%)	27 (7)	4 (6)	23 (7)	0.93

L/S, ratio of liver-to-spleen fat; HDL, high-density lipoprotein; LDL, low-density lipoprotein; AST, aspartate transaminase; ALT, alanine aminotransferase; γ-GTP, gamma glutamyl transpeptidase; ACEI, angiotensin converting enzyme inhibitor; ARB, angiotensin type 1 receptor blocker; DPP-4, dipeptidyl peptidase 4.

Characteristics of subjects according to the presence or absence of high-risk plaques are shown in Table 2. Compared with patients who did not have high-risk plaques, patients with high-risk plaques were older, more likely to be male, were currently smoking, and had hypertension, dyslipidemia, and/or type-2 diabetes mellitus, a lower level of HDL-cholesterol, and higher levels of triglyceride and hemoglobin A1c. With respect to medications, patients with high-risk plaques were more often prescribed an angiotensin-converting enzyme or angiotensin receptor blocker, statins, and α-glucosidase inhibitors and/or dipeptidyl peptidase-4 inhibitors. With regard to adiposity, patients with high-risk plaques had an above-average body mass index, waist circumference, and VAT as well as a greater prevalence of nonalcoholic hepatic steatosis.

**Table 2:** Characteristics of patients with and without high-risk plaques.

	Patients without high-risk plaques (n = 353)	Patients with high-risk plaques (n = 61)	p
Age (years)	63 ± 15	69 ± 9	<0.01
Men, n (%)	167 (47)	43 (70)	<0.01
Body mass index (kg/m^2^)	24 ± 4	25 ± 3	0.02
Waist circumference (cm)	84 ± 11	87 ± 8	0.01
Hypertension, n (%)	197 (55)	45 (74)	<0.01
Dyslipidemia, n (%)	161 (46)	37 (61)	0.03
Diabetes mellitus, n (%)	87 (25)	31 (51)	<0.01
Current smoking, n (%)	57 (19)	17 (32)	0.03
Total cholesterol (mg/dl)	192 ± 36	187 ± 37	0.38
Triglycerides (mg/dl)	108 (78)	136 (111)	<0.01
HDL-cholesterol (mg/dl)	59 ± 16	52 ± 13	<0.01
LDL-cholesterol (mg/dl)	114 ± 32	114 ± 33	0.9
Hemoglobin A1c (%)	6.1 ± 1.2	6.8 ± 1.6	<0.01
Subcutaneous adipose tissue (cm^2^)	146 ± 85	144 ± 64	0.86
Visceral adipose tissue (cm^2^)	89 ± 55	111 ± 47	<0.01
Nonalcoholic hepatic steatosis liver/spleen fat ratio <1.0	42 (12)	22 (36)	<0.01
Metabolic syndrome, n (%)	65 (18)	18 (30)	0.046
Medications			
ACEI or ARB, n (%)	120 (34)	29 (48)	0.04
Calcium channel blocker r, n (%)	99 (28)	22 (18)	0.19
Statin, n (%)	96 (27)	27 (44)	<0.01
Sulfonylurea, n (%)	11 (3)	5 (8)	0.06
α-glucosidase inhibitor, n (%)	7 (2)	10 (16)	<0.01
DPP4-inhibitor, n (%)	18 (5)	9 (15)	<0.01
Insulin, n (%)	22 (6)	5 (8)	0.56

L/S, ratio of liver-to-spleen fat; HDL, high-density lipoprotein; LDL, low-density lipoprotein; ACEI, angiotensin converting enzyme inhibitor; ARB, angiotensin type 1 receptor blocker; DPP-4, dipeptidyl peptidase 4.

**Fig 3:**
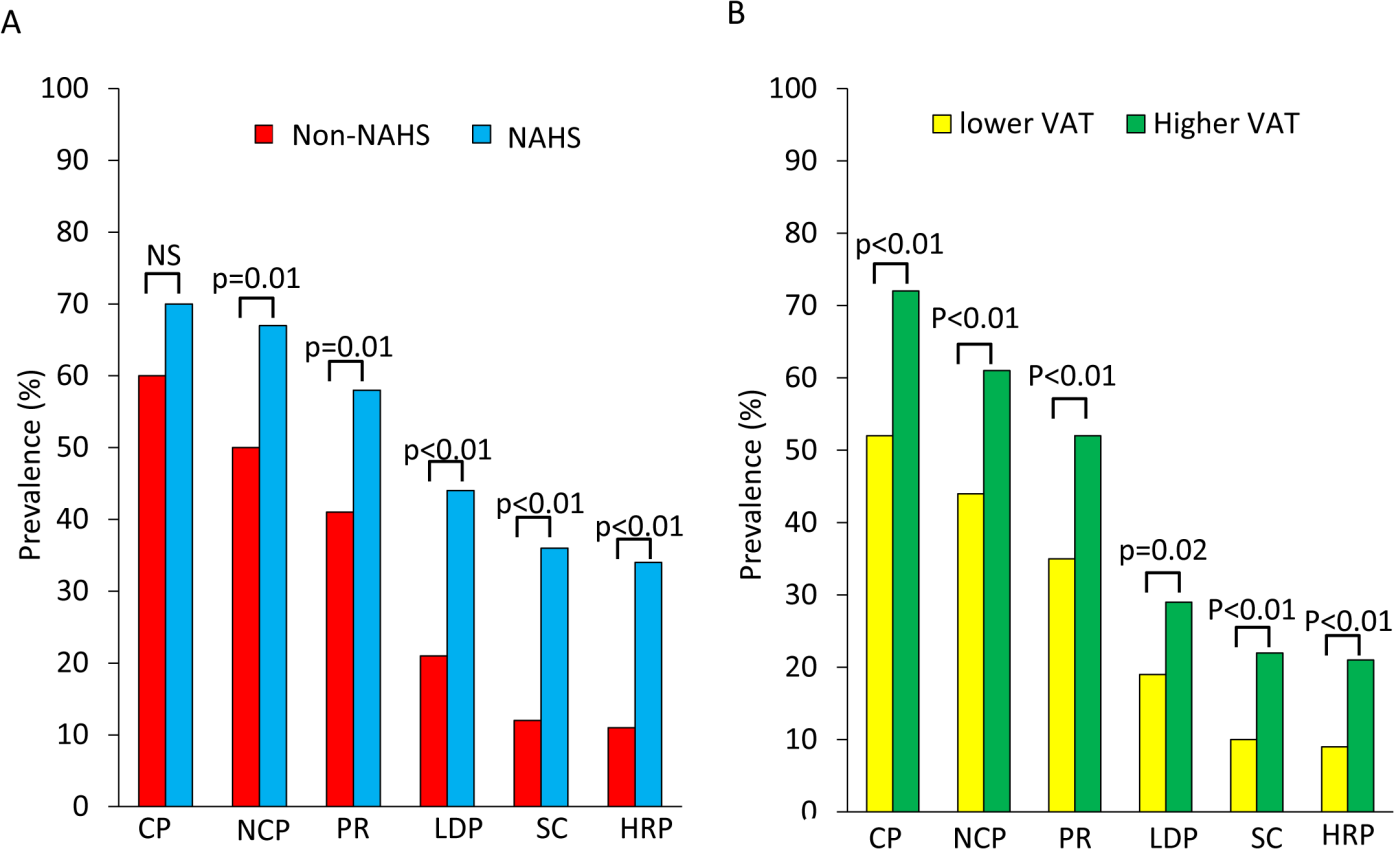
Characteristics of high-risk plaques. (A) Characteristics of high-risk plaques in patients with and without nonalcoholic hepatic steatosis and (B) in patients with higher and lower VAT scores (cutoff, 86 cm^2^). NAHS, nonalcoholic hepatic steatosis; NS, not significant; CP, calcified plaque; NCP, non-calcified plaque; PR, positive remodeling; LDP, low-density plaque; SC, spotty calcification; HRP, high-risk plaque.

Fig 3A shows the characteristics of plaques in patients with or without nonalcoholic hepatic steatosis. The percentage of patients with and without nonalcoholic hepatic steatosis who had calcified plaques was not significantly different (60% *vs*. 70%, p = 0.13). However, patients with nonalcoholic hepatic steatosis had significantly more non-calcified plaques than patients who did not have nonalcoholic hepatic steatosis (67% *vs*. 50%, p = 0.01), positive remodeling (58% *vs*. 41%, p = 0.01), low-density plaques (44% *vs*. 21%, p<0.01), spotty calcification (36% *vs*. 12%, p<0.01), and high-risk plaques (34% *vs*. 11%, p<0.01). Fig 3B shows the correlation between plaque characteristics and VAT accumulation. Patients with more VAT also had significantly more calcified plaques (72% *vs*. 52%, p<0.01), non-calcified plaques (61% *vs*. 44%, p<0.01), positive remodeling (52% *vs*. 35%, p<0.01), low-density plaques (29% *vs*. 19%, p = 0.02), spotty calcification (22% *vs*. 10%, p<0.01), and high-risk plaques (21% *vs*. 9%, p<0.01). A significant difference in the Agatston score was found for high- and low-VAT patients (37 (216) *vs*. 0.5 (151), p<0.01), but not for patients with and without nonalcoholic hepatic steatosis (25 (152) *vs*. 9 (192), p = 0.64).

Table 3 shows the number of coronary-artery plaques according to classifications of nonalcoholic hepatic steatosis and VAT. Patients with nonalcoholic hepatic steatosis had more non-calcified plaques (1.8±2.0 *vs*. 1.3±1.8, p = 0.03), low-density plaques (0.7±1.0 *vs*. 0.3±0.7, p<0.01), and high-risk plaques (0.5±0.9 *vs*. 0.2±0.5, p<0.01) than those who did not have nonalcoholic hepatic steatosis. Patients with higher VAT had more calcified plaques (3.1±3.2 *vs*. 1.9±2.7, p<0.01), non-calcified plaques (1.7±2.0 *vs*. 1.1±1.7, p<0.01), mixed plaques (1.2±1.7 *vs*. 0.7±1.2, p<0.01), and high-risk plaques (0.3±0.7 *vs*. 0.1±0.4, p<0.01) than those with lower VAT. No significant difference was found for coronary-artery stenosis when nonalcoholic hepatic steatosis and VAT classifications were compared.

**Table 3:** Plaque characteristics of patients according to NAFLD and VAT status.

	Nonalcoholic hepatic steatosis	Without nonalcoholic hepatic steatosis	p	Higher VAT	Lower VAT	p
Calcified plaque	3.0 ± 3.2	2.4 ± 3.0	0.19	3.1 ± 3.2	1.9 ± 2.7	<0.01
Non-calcified plaque	1.8 ± 2.0	1.3 ± 1.8	0.03	1.7 ± 2.0	1.1 ± 1.7	<0.01
Mixed plaque	1.2 ± 1.5	0.9 ± 1.5	0.21	1.2 ± 1.7	0.7 ± 1.2	<0.01
Low-density plaque	0.7 ± 1.0	0.3 ± 0.7	<0.01	0.4 ± 0.8	0.3 ± 0.7	0.18
High-risk plaque	0.5 ± 0.9	0.2 ± 0.5	<0.01	0.3 ± 0.7	0.1 ± 0.4	<0.01
Significant stenosis	0.4 ± 0.8	0.4 ± 1.0	0.97	0.4 ± 1.0	0.3 ± 0.9	0.41

VAT, visceral adipose tissue.

**Fig 4:**
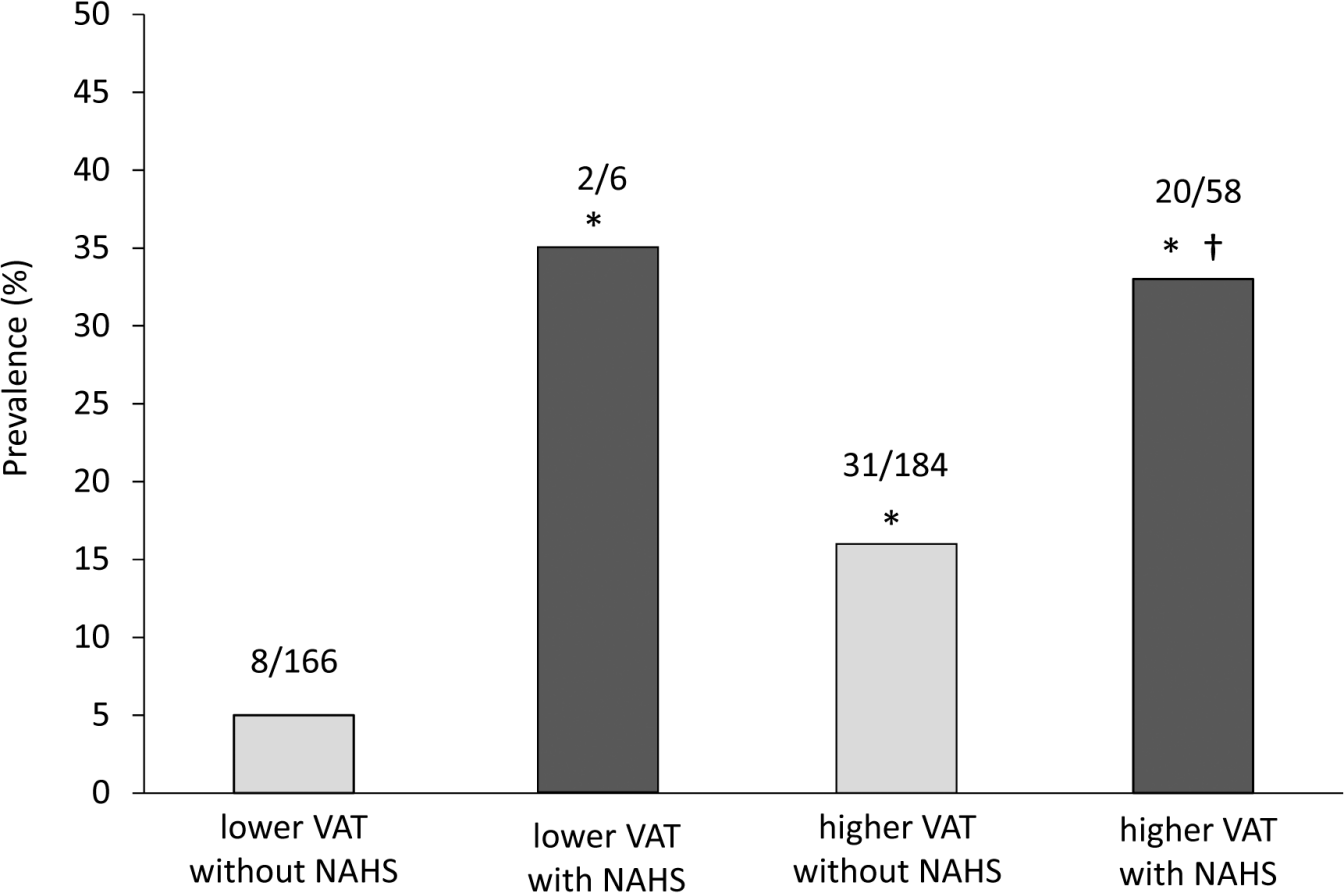
Prevalence of high-risk plaques according to presence or absence of nonalcoholic hepatic steatosis and the VAT area in the patients. The number of patients with high-risk plaques and total number of patients in each group is given above each bar. * p<0.05 *vs*. subjects without nonalcoholic hepatic steatosis with lower VAT. † p<0.05 *vs*. subjects without nonalcoholic hepatic steatosis with higher VAT. NAHS, nonalcoholic hepatic steatosis.

According to the univariate analysis, nonalcoholic hepatic steatosis was correlated significantly with high-risk plaques after patients had been adjusted independently for age, sex, hypertension, dyslipidemia, type-2 diabetes mellitus, smoking habits, as well as use of antihypertensive agents, statins, and hypoglycemic agents (Table 4). Multivariate analysis (which included nonalcoholic hepatic steatosis and VAT) suggested that nonalcoholic hepatic steatosis was associated independently with high-risk plaques (Table 4). Percentages of patients with high-risk plaques were 5, 16, 35, and 33 in patients who did not have nonalcoholic hepatic steatosis and lower VAT; patients who did not have nonalcoholic hepatic steatosis and higher VAT; patients with nonalcoholic hepatic steatosis and lower VAT; and patients with nonalcoholic hepatic steatosis and higher VAT, respectively (Fig 4). In patients with nonalcoholic hepatic steatosis, the percentage of patients with high-risk plaques was similar irrespective of VAT area.

**Table 4:** Odds ratios for high-risk plaques.

	Univariate		Multivariate	
Clinical characteristic	OR	P	OR	p
	(95% CI)		(95% CI)	
Age ≥65 years	2.82 (1.52–5.23)	<0.01	3.83 (1.80–8.14)	<0.01
Male	2.66 (1.48–4.79)	<0.01	1.94 (0.99–3.78)	0.05
Hypertension	2.23 (1.21–4.09)	<0.01	0.77 (0.33–1.76)	0.53
Dyslipidemia	1.83 (1.06–3.20)	0.03	0.80 (0.33–1.92)	0.61
Diabetes mellitus	3.16 (1.81–5.52)	<0.01	2.03 (0.84–4.91)	0.12
Currently smoking	2.01 (1.07–3.76)	0.03	1.99 (0.95–4.17)	0.07
Antihypertensive agent use	1.77 (1.00–3.12)	0.04	1.27 (0.59–2.73)	0.54
Statin use	2.13 (1.22–3.71)	<0.01	2.15 (0.88–5.23)	0.09
Hypoglycemic agent use	2.78 (1.53–5.08)	<0.01	1.11 (0.41–2.98)	0.84
Visceral adipose tissue >74 cm^2^	4.33 (2.13–8.79)	<0.01	2.24 (1.01–4.94)	0.046
Nonalcoholic hepatic steatosis liver/spleen fat ratio <1.0	4.18 (2.26–7.72)	<0.01	4.20 (1.94–9.07)	<0.01

OR, odds ratio; CI, confidence interval; NAFLD, non-alcoholic fatty liver disease Multivariate model includes age, gender, hypertension, dyslipidemia, diabetes mellitus, currently smoking, antihypertensive agent, statins, and and/or hypoglycemic agent use, higher visceral adipose tissue and nonalcoholic hepatic steatosis

Multivariate model includes age, gender, hypertension, dyslipidemia, diabetes mellitus, currently smoking, antihypertensive agent, statins, and and/or hypoglycemic agent use, higher visceral adipose tissue and nonalcoholic hepatic steatosis.

## Discussion

The main finding of the present study was a strong correlation between nonalcoholic hepatic steatosis and high-risk coronary-artery plaques independent of VAT according to multivariate logistic analyses. Nonalcoholic hepatic steatosis, therefore, is a valuable predictor of high-risk plaques.

NAFLD has been suggested to be a risk factor for CAD [2]. Non-diabetic patients with NAFLD have been shown to have decreased brachial artery endothelial flow-mediated vasodilation than those of matched controls [17]. A cross-sectional study showed a marked increase in carotid-artery intima-media thickness in NAFLD patients [18]. NAFLD has also been associated with increased calcification in the carotid arteries [3] and significant coronary stenosis as detected by invasive coronary angiography [19]. Hepatic steatosis has also been associated with calcification in coronary arteries and abdominal arteries. [20] NAFLD changes the balance between coagulation and fibrinolysis by causing (at least in part) atherothrombotic complications [21]. Those findings support a relationship between NAFLD and CAD.

Previously, we reported that large accumulation of VAT is an independent predictor of high-risk plaques as determined by CT angiography [11]. However, visceral obesity, insulin resistance, and the metabolic syndrome are also commonly associated with NAFLD, suggesting that cardiovascular risks for NAFLD patients could also be attributed to high VAT levels. Thus, NAFLD increases risk of type-2 diabetes mellitus and cardiovascular disease [22].

In the present study, nonalcoholic hepatic steatosis was diagnosed by CT, not by liver biopsy. Recent studies have shown that, in patients with biopsy-proven NAFLD, greater thickness of epicardial fat is associated with the severity of liver fibrosis [23]. The association between liver fibrosis with carotid/coronary atherosclerosis have also been reported in chronic infection with the hepatitis-C virus [24] [25], suggesting a link between chronic liver disease and cardiovascular disease [26].

Three reports have addressed the relationship between NAFLD and the characteristics of coronary plaques. Akabame and colleagues reported that plaques with lipid cores are independently associated with NAFLD after adjustment for covariates (including body mass index) for 298 patients suspected of having CAD [4]. A smaller study of 61 subjects reported that multivariate analyses (including the metabolic syndrome) showed a significant association between noncalcified plaques and grade of fatty liver [12]. Recently, Puchner and colleagues reported the results of the Rule Out Myocardial Ischemia/Infarction by Computer Assisted Tomography II trial [27]. That study showed that, in patients with suspected acute coronary syndrome, NAFLD was associated with high-risk coronary plaques independent of traditional cardiovascular risk factors as well as the extent and severity of CAD. Prevalence of features of high-risk plaques such as the “napkin-ring sign”, positive remodeling, low-density plaques, and spotty calcification in NAFLD patients was significantly greater than those in patients who did not have NAFLD. The results of the study by Puchner and colleagues are consistent with our findings, but their study did not include information on VAT. Our data suggest that the association between nonalcoholic hepatic steatosis and high-risk coronary plaques is independent of VAT and traditional cardiovascular risk factors.

The mechanism underlying the link between NAFLD and CAD is incompletely understood. NAFLD is associated with obesity, particularly with visceral obesity [2, 28]. However, NAFLD also has been shown to be associated with atherogenic dyslipidemia, hyperglycemia, and hypertension, regardless of the amount of visceral fat. Speliotes and colleagues reported that patients with NAFLD as detected by CT had higher average levels of triglycerides, lower levels of HDL-cholesterol, and higher levels of fasting glucose than those of healthy controls regardless of their visceral fat component [13]. Assy and colleagues showed that the presence of coronary stenosis in NAFLD patients was not increased if patients had metabolic syndrome. [12] That finding suggests that NAFLD may have a causal role in the development of atherosclerosis or that NAFLD may be associated with other unidentified risk factors for cardiovascular diseases. A potential mechanism by which NAFLD could cause an increase in cardiovascular risk is *via* increased levels of proinflammatory cytokines. Our study showed a significant difference in levels of high-sensitivity CRP for patients with nonalcoholic hepatic steatosis and in those who did not have nonalcoholic hepatic steatosis. Chronic, low-grade systemic inflammation may account for the increased prevalence of coronary plaques [29, 30]. In NAFLD, oxidative stress may induce production of tumor necrosis factor-α and interleukin-6 [31] and add atherogenic stimuli to the already high oxidative and proinflammatory status that is closely associated with metabolic syndrome. One study showed that levels of fetuin-A (which inhibits arterial calcification) were lower in patients with higher visceral fat thickness and was an independent predictor of NAFLD [32]. However, the direct effect of NAFLD on the morphology of atherosclerotic plaques was not evaluated in that study. Further studies are needed to ascertain the critical and specific factors linking between NAFLD and high-risk plaques.

Several population-based cohort studies have reported that NAFLD, as diagnosed by ultrasonography, is an independent predictor of cardiovascular events with an increased relative risk compared with a control population [33–35]. Our data demonstrate that patients with nonalcoholic hepatic steatosis have more low-density plaques and plaques characterized by positive remodeling and spotty calcification than those of patients who do not have nonalcoholic hepatic steatosis. Positive vascular remodeling, low-attenuation plaque content, and adjacent calcification in coronary plaques have been found to coexist in the culprit lesions of individuals with acute coronary syndrome [36, 37]. Taken together, the plaque characteristics in patients with nonalcoholic hepatic steatosis explain (at least in part) why they carry at an increased risk for CAD, though data are not sufficient to support a firm association between NAFLD and cardiovascular-related death.

In the present study, the prevalence of diabetes mellitus in patients with nonalcoholic hepatic steatosis was significantly higher than that in patients who did not have nonalcoholic hepatic steatosis. Nonalcoholic hepatic steatosis has been reported to be a cause of insulin resistance and diabetes mellitus [7], whereas diabetes mellitus itself is a critical factor for high-risk coronary plaques [38]. Our multivariate logistic analyses revealed that diabetes mellitus tended to predict high-risk plaques. The duration of diabetes mellitus (which was not documented in our study) is an important factor for alterations in coronary plaques. Precise analyses of the impact of diabetes mellitus on plaque morphology are lacking, whereas nonalcoholic hepatic steatosis apparently leads to an increased risk of development of both types of cardiovascular disease.

Our study had limitations. First, the cross-sectional design made determination of the causal or temporal relationships between nonalcoholic hepatic steatosis and high-risk plaques difficult. Prospective studies are required to confirm our findings and to assess potential interactions between NAFLD and high-risk plaques. Second, CT results may not be sufficient for the diagnosis of NAFLD. A liver biopsy has been the gold standard for the diagnosis of NAFLD [39] and histological data that would support the diagnosis of NAFLD were not documented in our study. However, less invasive diagnostic methods would be preferable. Unenhanced CT may aid the qualitative diagnosis of NAFLD [40]. Third, we excluded patients on oral corticosteroids and/or class-III anti-arrhythmic agents as well as those with a history of hepatic viral infection and alcohol consumption of ≤20 g per day. However, patients undergoing statin therapy or patients with dysthyroidism were not excluded. Inclusion of these patients might have affected our results. Even so, our multivariate analyses showed that nonalcoholic hepatic steatosis is an independent risk factor for high-risk plaque evens if patients were taking statins.

In conclusion, nonalcoholic hepatic steatosis, as assessed by the L/S attenuation ratio determined by MDCT, is strongly correlated with coronary artery plaques (especially high-risk plaques) independent of VAT. NAFLD may, therefore, be a relevant predictor of acute coronary syndrome.
